# Multidisciplinary treatment is necessary in glioblastoma with extracerebral metastases

**DOI:** 10.1007/s00066-024-02359-8

**Published:** 2025-01-22

**Authors:** Niklas B. Pepper, David R. Steike, Heidi Yppärilä-Wolters, Michael Müther, Dorothee Wiewrodt, Hendrik Berssenbrügge, Oliver Grauer, Philipp Lenz, Walter Stummer, Hans T. Eich

**Affiliations:** 1 Department of Radiation Oncology, University Hospital of Muenster, Albert-Schweitzer-Campus 1, Building A1, 48149 Muenster, Germany; 2 Department of Neurosurgery, University Hospital Muenster, Muenster, Germany; 3 Department of ear, nose and throat medicine, University Hospital Muenster, Muenster, Germany; 4 Department of Neurology with Institute of Translation Neurology, University Hospital Muenster, Muenster, Germany; 5 Department of Palliative Care, University Hospital Muenster, Muenster, Germany

**Keywords:** High-grade glioma, Radiotherapy, Radiochemotherapy, Extra-axial manifestation, Palliative care

## Abstract

**Purpose:**

While glioblastoma is the most common malignant brain tumor in adults, extracerebral manifestations are very rare in this highly aggressive disease with poor prognosis.

**Methods:**

We conducted a systematic literature review in the PubMed database and complemented the data by inclusion of a case treated in our clinic. In this context, we report on a 60-year-old woman with a right frontal glioblastoma, *IDH* wildtype, *MGMT* methylated.

**Results:**

Six months after initial diagnosis and primary treatment, there was extensive local intracranial progression with additional extension into the subcutaneous and frontotemporal cranial bones. Despite continuation of multimodal treatment, further extracerebral manifestations occurred 11 months after the initial diagnosis, both in the cranial bone as well as metastases in the right parotid gland, cervical lymph nodes, and lungs. While local radiotherapy enabled the cerebral lesions to be controlled, the patient’s clinical condition deteriorated rapidly despite simultaneous systemic therapy. The treatment had to be discontinued, and the patient died 5 weeks after confirmation of the multilocal extracerebral manifestations and a total of 12 months after initial diagnosis.

**Conclusion:**

Extracerebral manifestations of glioblastoma require close collaboration and joint decision-making with the patient, with an emphasis on palliative strategies.

## Introduction

Glioblastoma (GBM) is the most common malignant brain tumor in adults. It is known for its dismal prognosis with high rates of progression, even after aggressive treatment with resection, radiotherapy, and chemotherapy [1]. Tumor manifestations as well as progression can be tied to impaired quality of life (QoL) [2, 3].

Standard first-line treatment consists of resection (as far as possible without additional deficits) followed by adjuvant chemoradiotherapy. The latter comprises focal radiotherapy to an extended target volume with 60 Gy in 30 fractions in combination with oral chemotherapy with temozolomide [4, 5] or—in selected cases—a combination of temozolomide and lomustine (CCNU) [6, 7]. Additionally, hypofractioned radiotherapy (RT) concepts can be used for older patients or if palliative intent prevails, for example with 40 Gy in 15 fractions [8, 9]. For recurrent or progressive disease, no clear treatment standards have yet been established. While re-resection, re-irradiation, and chemotherapy also play a role in this situation, high-quality evidence is lacking, and long-term treatment effectiveness seems to be rare [1]. Since most recurrences appear at the site of primary treatment, radiotherapy is oftentimes finite due to the limited tolerability of adjacent organs at risk [10], with an as-yet unmet need to increase treatment effectiveness [11, 12]. CCNU is commonly used in second-line chemotherapy due to its solid performance in several trials [13]. Reports about extracerebral and spinal spread are rare and seem to be tied to poorer prognosis, although some studies report an increase in cases with systemic disease dissemination, potentially because of the increased survival rates achieved by modern primary treatment [14, 15].

## Clinical presentation of the available data

**Fig. 1:**
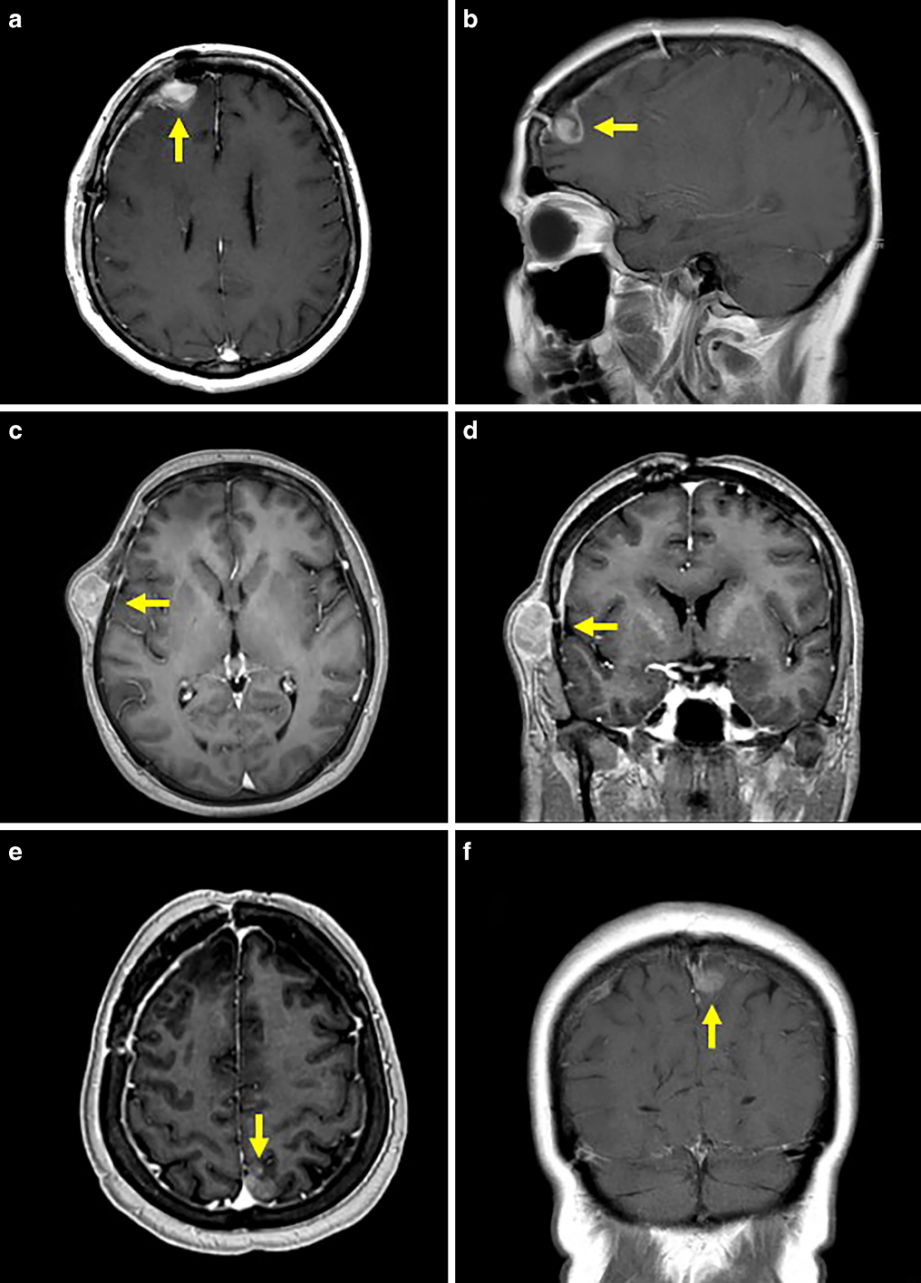
MRI scans of glioblastoma manifestations before systemic spread, in order of appearance: **a**, **b** initial manifestation with resection cavity localized in the right frontal lobe (arrow; postoperative imaging since preoperative imaging was not available); **c**, **d** first extracerebral manifestation in the left temporoparietal skull (arrow); **e**, **f** distant manifestation of the left occipital lobe (arrow)

A 60-year-old white female patient was referred to our radiation oncology department with a confirmed diagnosis of glioblastoma in the right frontal lobe (Fig. 1a, b) after primary resection. Primary treatment consisted of 60 Gy focal radiotherapy and concomitant temozolomide following the European Organisation for Research and Treatment of Cancer (EORTC) protocol [5], which was tolerated without any toxicity exceeding local alopecia and moderate fatigue. After three cycles of temozolomide, local progression in the right frontotemporal lobe with spread to the bone of the frontotemporal skull occurred (16 weeks after completion of radiotherapy). The bony lesion appeared as contrast enhancing in T1-weighted MRI and did not cause pain (Fig. 1c, d). It developed rapidly from clinically and radiographically unapparent to a maximal diameter of 3.0 × 1.7 × 2.7 cm in the span of 10 weeks. After resection of the subcutaneous manifestation, local re-irradiation with 39.6 Gy to the recurrent tumor site and concomitant CCNU (100 mg/m^2^ KOF d1, aiming for six cycles of 42 days) was administered.

**Fig. 2:**
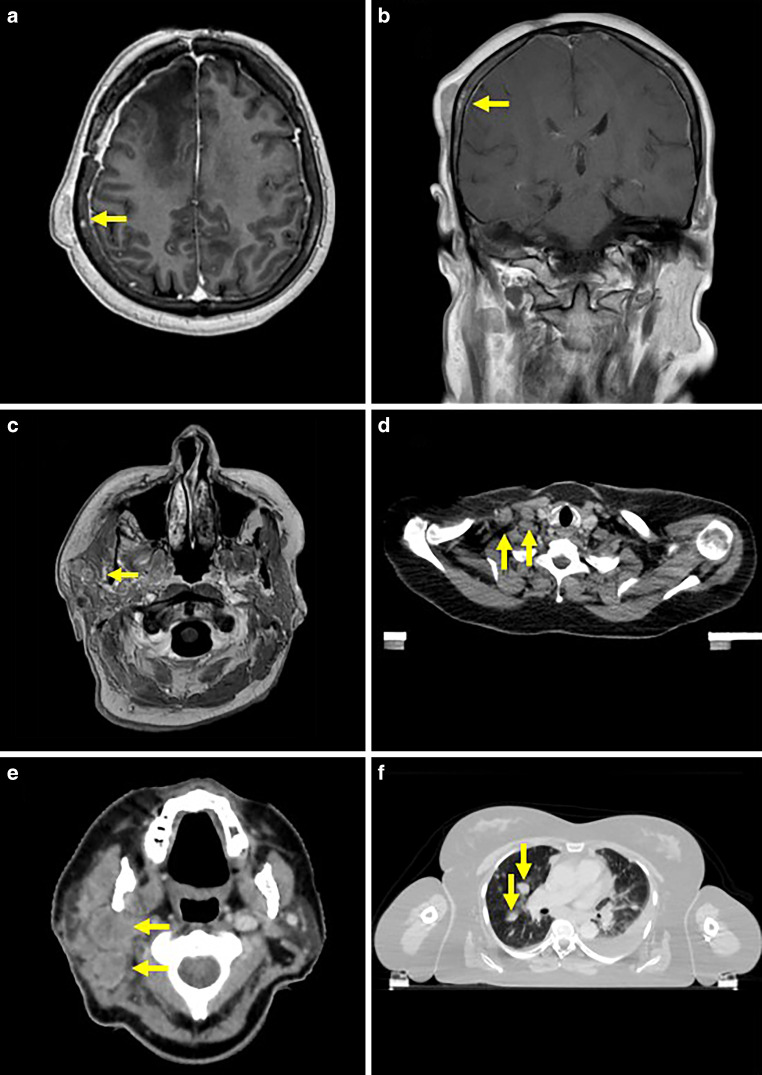
Imaging of systemic spread: MRI scans of the second glioblastoma manifestation in the right temporoparietal skull (**a**, **b**; arrow) and painful metastases in the right parotid (**c**; arrow) and CT scans of enlarged cervical lymph nodes (**d**, **e**; arrows) and pulmonary metastases (**f**; arrows)

After two cycles of CCNU (31 weeks after completion of primary radiotherapy), follow-up MRI revealed a new manifestation in the left occipital lobe (Fig. 1e, f). After thorough discussion of therapeutic options, the patient declined further resection but agreed to treatment with moderately hypofractioned radiotherapy (40 Gy in 15 fractions). Concurrent chemotherapy with CCNU was maintained. During the course of treatment, the patient noted painful swelling of the right parotid and two small sites of bulging located on the right temporal and parietal skull (Fig. 2a–c). MRI revealed nodal manifestations in the right parotid as the source of the pain, appearing as contrast enhancing and hyperintense in T1- and T2-weighted imaging. The bony lesions reassembled the manifestation in the frontotemporal skull, which was removed prior to second-line treatment. An additional CT scan of the head and neck area also revealed bilateral cervical lymphadenopathy in levels 2–5, with enlarged lymph nodes of up to 2.5 cm in diameter, as well as several bipulmonary nodes with a high probability of malignancy (Fig. 2d–f). The interdisciplinary neurooncology team as well as colleagues from the departments of ear, nose, and throat medicine and palliative care discussed the current oncological situation with the patient extensively. An extended resection was discussed and rejected by the patient after extensive information, based on the high risk of a deterioration in quality of life due to postoperative facial paralysis. Instead, partial resection/extended biopsy for diagnostic purposes and pain relief of the painful right parotid manifestation was performed. Histological assessment confirmed the suspected infiltration of glioblastoma.

Next-generation sequencing did not reveal a target for personalized therapy. Third-line systemic therapy with regorafenib was discussed, but eventually not administered due to deterioration of the patient’s physical status and resilience. While treatment was generally well tolerated, increasing fatigue and frailty became more and more apparent after multiple courses of chemo- and radiotherapy. Local radiotherapy with palliative intent to reduce pain and swelling was offered and accepted by the patient, also aiming to prevent painful regrowth. Unfortunately, 4 days after initiation, RT had to be discontinued due to rapid clinical deterioration which caused hospitalization in the palliative ward. Progressive respiratory impairment led to the diagnosis of pleural effusion, which could be controlled in terms of symptoms by pleurodesis. Cytology was negative for malignant cells.

At this point, in consent with the patient, all forms of tumor-directed treatment were discontinued. The patient died surrounded by relatives in a comforting environment with controlled symptoms.

Overall survival was 12 months after the initial diagnosis; in detail: 38 weeks after first confirmation of extracerebral manifestation in the right temporal skull, 7 weeks after second progression in the right occipital lobe, and 5 weeks after diagnosis and confirmation of parotid, cervical, and pulmonary systemic manifestation.

### Details regarding histopathology

**Fig. 3:**
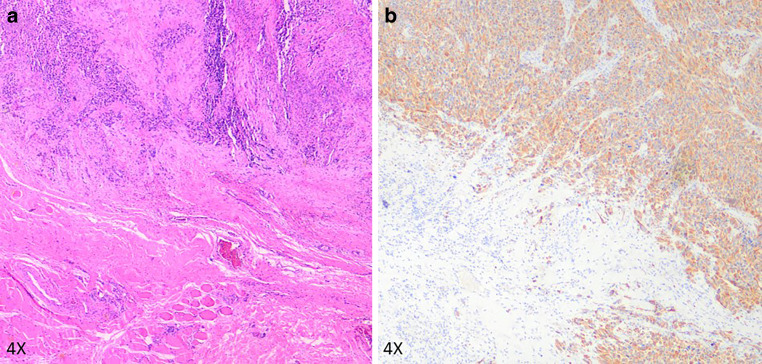
Histopathological HE (**a**) and GFAP (**b**) staining of the first extracerebral, subcutaneous manifestation at the right temporal skull. Images provided by Prof. Christian Thomas, Department of Neuropathology of the University Hospital Muenster

**Fig. 4:**
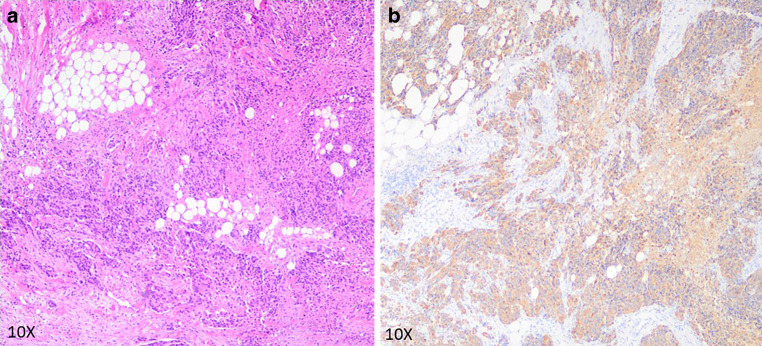
Histopathological HE (**a**) and GFAP (**b**) staining of the sequential extracerebral manifestation in the right parotid. Images provided by Prof. Christian Thomas, Department of Neuropathology of the University Hospital Muenster

The initial pathology report found a tumor of astrocytic differentiation with highly pathological blood vessel patterns infiltrating the adjacent structures (bone as well as connective tissue). Immunohistochemistry revealed positivity for GFAP and negativity for IDH1-mutation-specific staining (R132H) with preserved ATRX expression and H3 trimethylation. Nuclear accumulation of p53 was detectable in 32% of cells; the Ki67/MIB1 proliferation index was 53%. Targeted next-generation sequencing disclosed a C228T *TERT* promoter mutation and confirmed the *IDH1/2* wildtype status. Histopathologic assessment of the first extracerebral manifestation (right temporal skull; Fig. 3) also showed GFAP-positive, IDH1-negative tissue with preserved ATRX expression and H3 trimethylation and a Ki67/MIB1 index of 53%, confirming the diagnosis of extracerebral GBM. The parotid metastasis (Fig. 4) also did not differ from the two previously examined samples, except for slightly reduced p53 accumulation (12%) and Ki67/MIB1 index (52%).

## Literature review

A literature search was conducted in the NCBI PubMed database using the search term “(Glioblastoma [All] OR Glioblastoma [MeSH Terms]) AND ((spreading [All] OR metastases [All] OR manifestation [All]) AND (extra [All] AND (cranial [All] OR cerebral [All] OR axial [All] OR neural [All]))).” All entries were screened for availability of the abstract in English and accessible abstracts were then reviewed. Results not fitting the subject at hand were excluded, for example if the article did not feature extracerebral metastases of glioblastoma. The remaining articles were all assessed regarding the timeline of metastatic development and survival data. The last date of literature review was 23.08.2024.

**Fig. 5:**
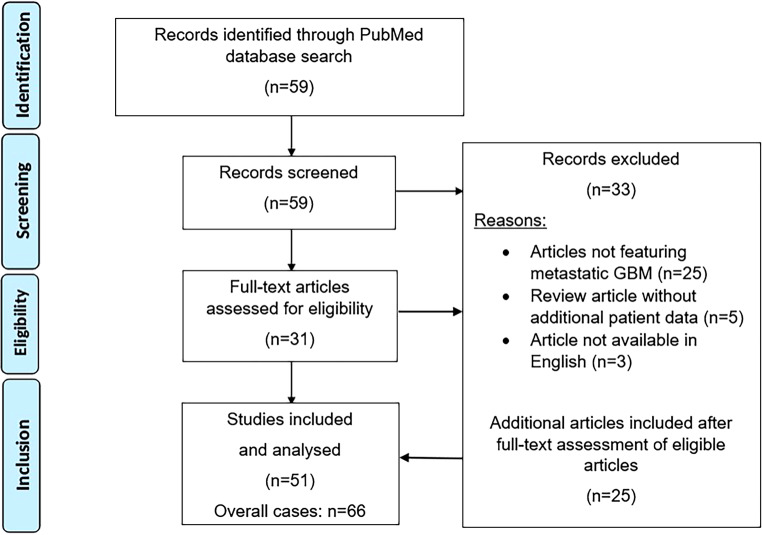
Preferred Reporting Items for Systematic reviews and Meta-Analyses (PRISMA) diagram of the performed literature review in the NCBI PubMed database

Our search strategy identified 59 results with the designed search string. All records were screened; 33 articles were excluded because they did not provide data on the subject. Cases identified during the subsequent review of full-text articles were also incorporated into the analysis. Figure 5 illustrates the reviewing workflow as well as details on exclusion as a Preferred Reporting Items for Systematic reviews and Meta-Analyses (PRISMA) flow diagram. All fitting cases are summarized in Table 1.

**Table 1:** Literature data: results of a comprehensive literature search on extracerebral manifestations of glioblastoma

Author	Case number	Primary location	Site of metastasis	TTM (months)	OS (months)	Reference
Potter et al. 1983	1	L frontal	Oss	11	< 1	[16]
Trattnig et al. 1990	1	L frontal	Oss	19	14	[17]
Wallace et al. 1996	1	R frontal	LN	N/A	< 1	[18]
Forsyth et al. 1998	1	R frontal	Scalp, LN	5	< 1	[19]
	2	R frontal	Scalp, LN	5	2	
Beauchesne et al. 2000	1	R temporal	Oss, pul	8	< 1	[20]
Houston et al. 2000	1	L parietal	Oss	16	1	[21]
	2	L temporal	Scalp	6	6	
	3	Bifrontal	LN	17	9	
Hübner et al. 2001	1	R cerebellum	LN	10	7	[22]
Allan et al. 2004	1	N/A	Scalp	12	2	[23]
Ogungbo et al. 2005	1	L occipital	LN, parotid, pul	12	2	[24]
Rajagopalan et al. 2005	1	R temporal	Oss	18	1	[25]
Taha et al. 2005	1	L frontal	LN, parotid	6	3	[26]
Didelot et al. 2006	1	N/A	Oss, pul	0	3	[27]
Mujic et al. 2006	1	L frontal	Pul, ST	25	1	[28]
Toledano Delgado et al. 2006	1	R temporal	Oss	10	N/A	–
Saad et al. 2007	1	L frontal	Scalp, pul, hep	6	4	[29]
Piccirilli et al. 2008	1	R frontal	Pul	16	9	[30]
	2	R temporal	Hep	18	8	
	3	L frontal	Oss	19	11	
	4	R occipital	Pul	21	10	
	5	R temporal	Pul	19	10	
	6	R frontal	LN	20	10	
	7	R temporal	Pul	13	12	
Templeton et al. 2008	1	L frontal	Oss, pul	5	6	[31]
	2	R frontal	Pul	24	24	
Zhen et al. 2010	1	R frontal	LN, oss	2	N /A	[32]
Armstrong et al. 2011	1	L frontal	Scalp	N/A	N/A	[33]
Beauchesne et al. 2011	1	R temporal	Oss, pul	1	N/A	[34]
	2	L temporal	Pul, hep	N/A	N/A	
Blume et al. 2012	1	R parietal	Oss, pul	36	N/A	[35]
Dawar et al. 2012	1	R temporal	Scalp, parotid, (pul)	54 (57)	6.5 (3)	[36]
Kalokhe et al. 2012	1	R temporal	Oss, pul	10	9	[37]
Guo et al. 2012	1	Pons	Scalp	8	N/A	[38]
Seo et al. 2012	1	L frontal	LN	60	6	[39]
Romero-Rojas et al. 2013	1	L frontal	LN, parotid, oss	6	18	[40]
Anghileri et al. 2015	1	L central	LN	82	1.5	[41]
Undabeitia et al. 2015	1	R temporal	Pul	5	3	[42]
Franceschi et al. 2016	1	N /A	Oss, pul, hep	N/A	N/A	[43]
Karatas et al. 2016	1	R temporal	LN, pul, oss	48	24	[44]
Lewis et al. 2017	1	L cerebellum	ST	N/A	N/A	[45]
Simonetti et al. 2017	1	L parietal	Pul, oss	48	2	[46]
Hori et al. 2018	1	Bifrontal	LN, pul	2.8	< 1	[47]
Janik et al. 2019	1	R temporal	Pul, muscle	19	3.5	[48]
Ruff et al. 2019	1	L temporal	Muscle	N/A	4	[49]
Swinnen et al. 2019	1	R temporal	LN, parotid, pul	6	8.5	[50]
Tamai et al. 2019	1	R temporal	Pul	N/A	N/A	[51]
Hsu et al. 2020	1	R temporal	Oss	15	N/A	[52]
Liu et al. 2020	1	L temporal	Scalp, (pul)	6 (18)	13 (2)	[53]
Rossi et al. 2020	1	R frontal	LN, oss	39	12	[54]
Umphlett et al. 2020	1	L occipital	LN, oss, pul, hep, ST	1	N/A	[55]
Denier et al. 2021	1	N/A	Pul, hep, ren	3	9	[56]
	2	N/A	Oss	3	15	
Sickler et al. 2021	1	R temporal	Oss	12	N/A	[57]
Al-Sardi et al. 2022	1	R parietal	LN, pul	59	N/A	[58]
Hersh et al. 2022	1	L parietal	Oss	10	N/A	[59]
Kumaria et al. 2022	1	L temporal	Pul	17	N/A	[60]
Nakib et al. 2022	1	R central	Scalp	6	N/A	[61]
Almeida et. al 2023	1	R frontal	Oss	3	N/A	[62]
	2	R frontal	Pul, hep	8	< 1	
	3	R frontal	Scalp	9	5	
Wang et al. 2023	1	R temporal	Scalp	2	N/A	[63]
Heinig et al. 2024	1	R temporal	LN, oss	7	N/A	[64]
Yuen et al. 2024	1	L frontal	Pul	8.5	1.5	[65]
	2	L cerebellum	Oss	4	5	

If information about the sequence of metastatic development was provided, corresponding sites and survival values (TTM and OS) are indicated by brackets

*L* left, *R* right, *hep* liver metastases, *N/A* data not available, *LN* lymph node metastases, *oss* bone metastases, *pul* lung metastases, *ren* kidney metastases, *ST* abdominal soft tissue, *TTM* time to metastases from primary diagnosis, *OS* overall survival (after diagnosis of metastases)

With our search strategy, we identified an overall number of 66 reported cases with extracerebral metastases of glioblastoma. The most common sites were the lung (31 cases) and bones (25 cases), followed by the lymph nodes (18 cases) and scalp (12 cases). Rarer sites were the liver (7 cases), parotid (5 cases), abdominal soft tissue (3 cases), muscle (2 cases), and kidney (1 case).

Survival data were available in 71% of cases. The median OS after initial diagnosis was 22.6 months (before 2000: 14.2 months; 2000–2009: 20.5 months; 2010–2019: 29.2 months; after 2019: 16.6 months). After the initial diagnosis, metastases occurred on average after 16 months (before 2000: 10.0 months; 2000–2009: 13.7 months; 2010–2019: 26.1 months; after 2019: 12.5 months). The median OS after the development of metastases was 6 months (before 2000: 3.5 months; 2000–2009: 6.6 months; 2010–2019: 6.9 months; after 2019: 6.2 months).

## Discussion

While glioblastoma is the most common malignant brain tumor in adults, presentation of extracerebral metastases is very rare. Swinnen et al. reported the very similar case of a 56-year-old female with temporal glioblastoma developing painful ipsilateral parotid metastases as well as cervical lymphadenopathy and lung metastases after first-line treatment [50]. Herein, cervical and pulmonary manifestations were also histologically verified as GBM, and additional target therapy was administered (avelumab + axitinib), resulting in an overall survival of 14.5 months after initial diagnosis.

Liu et al. describe the case of a male patient developing ipsilateral scalp metastases and consequent lung metastases after initial treatment [53]. In their additional review of the literature, the authors also identified 12 similar cases of patients with multiple extracerebral metastases in which the cervical lymph nodes, bones, lungs, and parotid were the predominant sites. The overall survival of the reported cases covers a wide range from weeks to years after initial diagnosis, but, just as in our case, patients with lung metastases seem to have the poorest prognosis [66].

Surprisingly, metastatic sites do not seem to differ substantially in terms of survival outcome: our literature research suggests that the survival of patients with development of lymph node metastases (mean OS: 7 months) or scalp metastases (mean OS: 5 months) was similar to patients with metastases in organs such as lung (mean OS: 6 months) or bone (mean OS 6 months). Analyzing the development of survival parameters, the increase over the past decades seems to be encouraging. Unfortunately, the lack of data and treatment regimens of the reported cases does not allow any robust conclusions on the reasons for the apparent deterioration of OS and TTM in the current decade. One reason could be the redefinition of glioblastoma in the WHO classification in 2021. Furthermore, the lack of information in the majority of reported cases does not allow for an interpretation of the impact of metastases on survival: it mostly remains unclear whether patients died as a result of intra- or extracranial progression. In our case, the cause of death was attributed to cerebral progression. It should be pointed out that the systemic metastases (while not lethal) were associated with a severe decline in quality of life based on substantial pain and shortness of breath.

While some of the reported cases in part lack a clear definition of the timeline as well as survival data, several authors have reported the development of local metastases on the scalp, in cranial bone, and in parotid or local lymph nodes before further systemic spread (e.g., to the lungs, bones, or liver) was detected in short succession, consistent with the development in our case [36, 53]. Therefore, early detection of metastatic sites and adjustment of systemic and local therapy seem necessary. However, there is no consensus on either optimal systemic treatment or the combination of chemotherapy, targeted therapy, and radiotherapy, nor are there recommendations for full-body staging in this rare situation. Swinnen et al. also highlight the need for radiological awareness when assessing follow-up imaging [50]. Especially with increasing numbers of long-time survivors, early detection of unusual manifestations could be crucial, and systemic staging seems to be appropriate in cases in which extracerebral manifestations are suspected.

The mechanism of dissemination has been discussed severalfold. Interestingly, while an iatrogenic component due to craniotomy has been discussed, several authors provide evidence of extracerebral spread without prior craniotomy [53, 67] and even at first manifestation [68–70]. As cervical lymph node metastases, as well as lung metastases and bone metastases, are the predominant manifestations, lymphatic and hematogenous pathways seem possible. Müller et al. found a rate of 20.6% of GBM patients with circulating tumor cells in peripheral blood [71], which is plausible since GBM cells are in contact with the circulatory system and the blood–brain barrier is impaired at tumor sites. Chen et al. demonstrated transmission of GBM which resulted in pulmonary and lymphatic metastases after lung transplant from a donor with GBM, but without known systemic manifestations [72]. This supports the hypothesis that extracerebral dissemination might be more common than expected but not frequently diagnosed, for example due to short survival periods [71]. Whether or not circulating tumor cells are possible vectors and why the rate of diagnosed extracerebral metastases (around 0.4–0.5% [73]) is still substantially below the demonstrated 20.6% of patients positive for circulating tumor cells is yet to be determined. In this context, prolonged immunosuppression (e.g., because of pre-existing conditions, long-term corticosteroid intake, or other reasons such as transplant medication, as indicated in the case report by Chen et al. [72]) might also be a factor promoting systemic spread [73]. Whether or not specific molecular alterations might be responsible for a higher tendency for systemic spread also remains unclear based on the accessible data. The demonstrated case did not show any unusual histopathological mutations in the workup, including next-generation sequencing. Due to the high variety of information provided in the compiled cases of this analysis, no conclusive evidence on the impact of molecular characteristics such as *MGMT* promotor methylation, *IDH* mutation status, or (epi-)genetic findings can be given, since specifics are reported infrequently. Future scientific effort to gather and analyze histopathologic material for detailed analysis of such cases of extracerebral metastases of GBM might reveal interesting insights into the pathomechanisms as well as optimal treatment, which remain elusive at the moment.

As highlighted by our case as well as by a recent case report by Heinig et al. [64], symptom control might be the primary need of patients with metastatic GBM, as extracerebral manifestations seem to oftentimes result in noticeable impairment of quality of life (painful metastases in the bones or parotid, impaired breathing due to lung metastases). Discussing the potential benefit and possible side effects of any form of treatment is a delicate matter when treating patients with recurrent or metastatic glioblastoma. Proper information provided by physicians delivering tumor-directed therapy (i.e., surgeons, radiation oncologists, and neurooncologists) should be accompanied by early integration of palliative care structures and psycho-oncological support for patients and relatives to help them reach a decision at each step along the way [74]. In our case, the close cooperation between all named disciplines allowed for the successive treatment de-escalation from aggressive radiochemotherapy to palliative local RT to best supportive care without any tumor-directed therapy—driven entirely by a patient empowered to make informed decisions.

## Conclusion

Extracerebral metastatic glioblastoma is rare and often only discovered at an advanced stage with severe clinical abnormalities. In addition to neurological problems, painful swelling of the skin in the head and face area, parotid gland and bone pain, or pulmonary symptoms can occur and significantly impair quality of life. The whole treating team should be aware of this rare form of the disease. If such disseminated intra- and extracranial seeding occurs, a careful interdisciplinary evaluation of treatment options and joint decision-making with and for the patient should take place, with an emphasis on palliative strategies.

## Data Availability

Please contact Niklas Benedikt Pepper regarding data availability.
